# Genome-Wide Analysis of *PP2C* Gene Family and Identification of *DlPP2C1* as an ABA-Responsive Candidate Regulator During Early Somatic Embryogenesis in Longan (*Dimocarpus longan* Lour.)

**DOI:** 10.3390/plants15111659

**Published:** 2026-05-28

**Authors:** Muhammad Awais, Hafiz Muhammad Usman, Xiaoqiong Xu, Chunyu Zhang, Yukun Chen, Shengcai Liu, Yuji Huang, Xu XuHan, Muniba Shafiq, Yuling Lin, Zhongxiong Lai

**Affiliations:** 1 Institute of Horticultural Biotechnology, Fujian Agriculture and Forestry University, Fuzhou 350002, China; awais9518@gmail.com (M.A.); muhammadusman@fafu.edu.cn (H.M.U.); xuxq0921@163.com (X.X.); zcynhba@163.com (C.Z.); cyk68@163.com (Y.C.); lshc9264@fafu.edu.cn (S.L.); yjhuang2004@163.com (Y.H.); munibashafiq@fafu.edu.cn (M.S.); buliang84@163.com (Y.L.); 2 Key Laboratory of Genetics, Breeding and Multiple Utilization of Crops, Ministry of Education, Fujian Agriculture and Forestry University, Fuzhou 350002, China; 3 Institut de la Recherche Interdisciplinaire de Toulouse, IRIT-ARI, 31300 Toulouse, France; xxuhan@163.com

**Keywords:** *Dimocarpus longan*, somatic embryogenesis, protein phosphatase 2C, ABA signaling

## Abstract

PP2C (protein phosphatases 2C) are key regulators of abscisic acid (ABA) signaling that play a crucial role in plant stress responses. Our analysis identified 71 *DlPP2C* genes in *Dimocarpus longan*, which were classified into distinct subgroups based on phylogenetic relationships with *Arabidopsis thaliana* and *Oryza sativa*. Structural analysis demonstrated conserved motif composition and gene organization within subgroups, while chromosomal distribution and synteny analysis revealed that segmental duplication events contributed to the expansion of this gene family. Promoter analysis uncovered several *cis*-acting elements related to hormone and stress responsiveness, especially abscisic acid-responsive elements (ABREs), suggesting that *DlPP2C* genes may play a role in ABA signaling pathways. Furthermore, we examined the ABA-responsive expression profiles of *DlPP2C* genes under exogenous ABA treatments. The expression patterns were dynamic and dose- and time-dependent, with several genes showing peak expression at 10 μM ABA after 16 h. The *DlPP2C1* in particular displayed a strong transcriptional response, indicating its potential role in ABA regulation. While overexpression and GUS staining assays revealed enhanced activity under ABA treatment, further supporting the involvement of PP2C in ABA-responsive regulation, further mechanistic studies are needed for a full characterization. Finally, RNA sequencing analysis revealed a total of 1799 differentially expressed genes in response to ABA, with a prevalence of downregulated genes, showing extensive transcriptional reprogramming. Functional enrichment analysis demonstrated that these genes were largely associated with plant hormone signaling, stress response, and metabolic pathways. Weighted gene co-expression network analysis revealed a total of 32 key gene modules associated with ABA signaling. Collectively, our findings propose that *DlPP2C* genes, especially *DlPP2C1*, play a key role in ABA-mediated regulatory networks and provide valuable insights into stress adaptation mechanisms, especially during early somatic embryogenesis in longan.

## 1. Introduction

Protein phosphatase 2C (PP2C), commonly known as metal-dependent-type protein phosphatases, has serine or threonine as its dephosphorylation site. In comparison to other protein phosphatases, the regulatory subunits are absent in PP2C, making it a monomeric enzyme whose activity depends on Mg^2+^ or Mn^2+^. PP2C may lose its activity when Mg^2+^ or Mn^2+^ are replaced with other ions such as CA^2+^ and Zn^2+^ [1]. The initial report on the *PP2C* gene family was published in relation to *Arabidopsis thaliana* and *Oryza sativa* in early 2008. According to that report, a total of 80 and 90 *PP2C* genes were found in *Arabidopsis thaliana* and *Oryza sativa*, respectively [2].

*Longan*, also known as “dragon’s eye” or *Dimocarpus longan*, is a tropical evergreen tree indigenous to Southeast Asia, particularly southern China, Taiwan, and Vietnam. It is closely related to *Lychee* and *Rambutan* and is a member of the *Sapindaceae* family. Significant progress has been made in the molecular research of *D. longan*, mainly due to genome sequencing initiatives [3]. In longan, somatic embryogenesis has been investigated thoroughly as a model system for regenerating woody plants. To identify the molecular mechanisms underlying embryo development, research has focused on molecular biology and proteomics during somatic embryogenesis, employing methods such as gene cloning, real-time quantitative PCR, and two-dimensional electrophoresis [4,5]. Furthermore, studies on the *PP2C* gene family have demonstrated its functional significance and evolutionary conservation across several plant species [6]. Techniques for tissue culture have been crucial to the conservation and multiplication of *D. longan*. These techniques benefit the mass propagation of superior genotypes and conserve cultivars at risk of extinction [7,8].

Previously published studies have shown that the conserved catalytic domains of PP2C in plants are mostly centered at the C terminus, whereas the N terminus serves as an extension zone with weak conservation and varying lengths, resulting in the different functioning of PP2C [9,10]. The clade A PP2C in *Arabidopsis* serves as a negative regulator in ABA signal transduction pathways. For instance, the *AtABI1* (a clade A PP2C member) has a conserved sequence at its C terminus, which forms a ternary complex with the ABA receptor (PYL) and SnRK2 kinases, thereby modulating downstream stress activities [11].

Genome sequencing and various bioinformatics software have provided basic yet powerful tools for the identification and analysis of gene families. At present, a large number of gene families have been identified and characterized at the whole-genome level, including TF families in *Dimocarpus longan*, including (bZIP, B3, ARF, ABI, MYB and NAC) [12,13,14,15,16]. *PP2Cs* are well organized as key negative regulators in core ABA signaling pathways, modulating the activities of downstream kinases and transcription factors to fine-tune plant responses to environmental stimuli. Although *PP2C* gene families have been extensively characterized in several model crop species, including grape (*Vitis vinifera* L.) [17], litchi (*Litchi Chinensis* Sonn.) [18], cucumber [19], strawberries (*Fragaria ananassa*) [20], peanut (*Arachis hypogaea*) [21] and soyabean (*Glycine max*) [22], but their deification and functional roles in *Dimocarpus longan* remain largely unexplored.

Here, a total of 71 *DlPP2C* genes were identified, and a comprehensive genome-wide analysis was performed to investigate their structural features, evolutionary relationships, and expression patterns. Furthermore, we functionally characterized *DlPP2C1* using transient overexpression and GUS assays and integrated transcriptomic analysis to elucidate its role in the ABA-responsive regulatory cascade. Our findings provide new insights into the molecular mechanisms underlying ABA signaling during early somatic embryogenesis in *longan* and establish a foundation for future studies on stress adaptation and genetic improvement of this economically important fruit crop.

## 2. Results

### 2.1. Physicochemical Properties Analysis of DlPP2C Gene Family

Based on the physicochemical properties, the structural features, and possible functional properties of the 71 PP2C proteins detected in *Dimocarpus longan* were analyzed. These were the amino acid length, molecular weight (MW), isoelectric point (pI), instability index (II), aliphatic index (AI), grand average of hydropathicity (GRAVY), and predicted subcellular localization (Table 1). The sizes of the DlPP2C proteins differed significantly, ranging from 155 amino acids (*DIPP2C54*) to 1018 amino acids (*DIPP2C69*), with a molecular weight of 17,163.21 Da to 28,459.06 kDa, respectively. Most plant proteins are usually in the 10–100 Da diameter range; however, because of its comparatively large size, *DIPP2C69* might have a more complicated structure, possibly enclosing more functional domains or regulatory regions than smaller proteins like *DIPP2C33*. The estimated isoelectric points were between 4.83 (*DIPP2C52*) and 8.91 (*DIPP2C34*), indicating a high variation in the charge features of the proteins. The *pI* values of most of the DlPP2C proteins were below 7, implying that they are mainly acidic. This property could enable them to be soluble and exercise their activity in intracellular organelles like the chloroplasts and the cytoplasm. The instability index was used to estimate protein stability, which varied significantly among *DlPP2Cs*, with a range of 31.26 (*DlPP2C5*) to 65.62 (*DlPP2C30*). Proteins that have an instability index above 40 are normally regarded as unstable, and therefore, a significant percentage of DlPP2C proteins might be less stable in vitro. Conversely, the aliphatic index (AI), a measure of the volume of aliphatic side chains (a measure of stability), ranged between 68.07 (*DIPP2C1*) and 101.12 (*DIPP2C41*). It is worth noting that a higher AI value is an indicator of increased structural stability, which means that a portion of DlPP2C proteins can maintain stability in different environmental conditions despite increased instability index. The GRAVY values were above −0.579 (*DIPP2C5*) and below 0.013 (*DIPP2C56*). The existence of negative GRAVY values implies that the majority of the proteins of DlPP2C are hydrophilic, which is a property commonly linked with soluble proteins that have a role in intracellular signaling. This finding is in line with the overall functional role of PP2C proteins in the plant signaling pathways. Subcellular localization prediction revealed that DlPP2C proteins are spread throughout various cellular compartments, such as the nucleus, cytosol, chloroplasts, mitochondria, vacuoles, and the plasma membrane. Such a large-scale localization pattern indicates that the DlPP2C proteins are involved in numerous biological processes, primarily in signal transduction and stress response, as well as metabolic processes.

### 2.2. Evolutionary Analysis of the PP2C Gene Family

**Figure 1:**
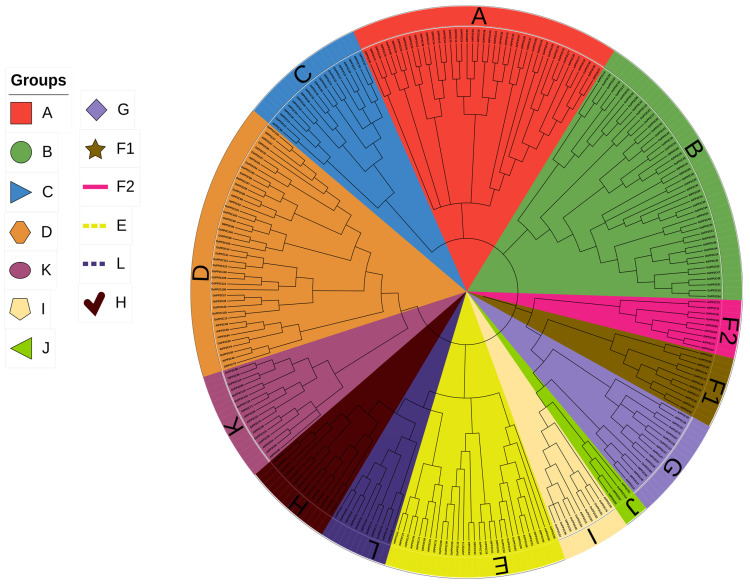
The phylogenetic tree of *PP2C* gene family in three species, including *D. longan*, *Arabidopsis thaliana*, and *Oryza sativa*. The tree is divided into various clades (A–L), which consist of a unique set of *PP2C* gene family members of the species, displayed in different colors.

In order to render the patterns of evolution and possible diversification of the *DlPP2C* gene family in terms of functionality, a phylogenetic tree was built using the multiple sequence alignment of the conserved *PP2C* catalytic domain. PP2C proteins of *Dimocarpus longan*, *Arabidopsis thaliana*, and *Oryza sativa*, belonging to dicot and monocot species, were analyzed. According to the phylogenetic topology, these PP2C proteins were divided into various clades (A–L). Each clade has a distinctive color, which indicates the evolutionary divergence of the proteins and their evolutionary subgroups (Figure 1). *D. longan* contains 71 *PP2C* genes, which is similar to the 80 reported members in *A. thaliana* but significantly lower than the 90 reported members in rice. The phylogenetic tree also revealed that the major clades did not have an even number of *DlPP2C* members. Clade A comprised the most members, 25 *DlPP2C*, and clade D had 1 *DlPP2C*. Clades F1 and F2 comprised four proteins of the *DlPP2C* group, and one of them was the smallest, consisting of one *DlPP2C* protein. Interestingly, *DlPP2C* was found in all the clades (A–L), and there was no significant decrease in *PP2C* in longan. The DlPP2C proteins within a clade were further clustered with their homologs of either *A. thaliana* or *O. sativa*, indicating that the large subfamilies of *PP2C* are very conserved across dicot and monocot species. This conserved phylogenetic distribution suggests that the split of *PP2C* genes probably preceded the split of those plants’ lineages. The fact that all major subgroups have representatives in longan is another argument in favor of the functional conservativity of PP2C proteins. On the whole, phylogenetic connections give important information on the evolutionary conservation and possible functional similarity of DlPP2C proteins with their homologs in model plant species.

### 2.3. PP2C Gene Clustering and Distribution of Chromosomes in Longan

**Figure 2:**
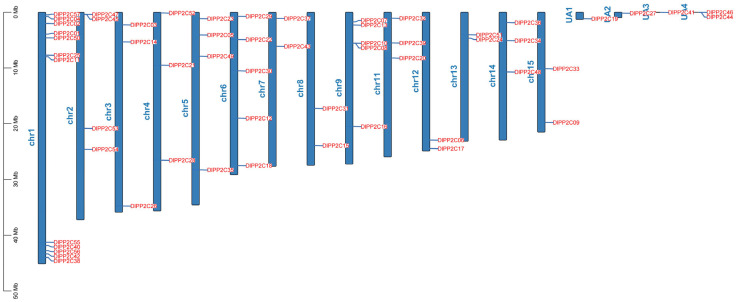
The chromosomal distribution of the *DIPP2C* gene family, where 15 chromosomes (Chr1-Chr 15) are clearly defined, with their genes being positioned in different locations on the chromosome. Unanchored (UA) regions contain additional *DIPP2C* genes, which is an indication that they are unassembled or that their genomic regions remain unresolved. The resulting diagram shows the physical locations of each family member (renamed according to standard homology-based nomenclature), illustrating that these evolutionarily related genes are distributed across multiple chromosomes.

After the discovery of the *PP2C* gene family, 71 possible *DlPP2C* genes have been mapped onto the *longan* genome and systematically renamed according to their position in the chromosome and physical order, from *DlPP2C1* to *DlPP2C7*1. The genes of *DlPP2C* were unevenly distributed in the chromosomes, and there were local instances of gene clustering. It is worth noting that *DlPP2C* genes were evenly spread in various chromosomes, even though their abundance was extremely uneven. A total of fifteen *DlPP2C* genes were found on chromosome 1, whereas only two *DlPP2C* genes were observed on chromosome 15. Moreover, there were 2 *DlPP2C* gene members in chromosomes 3, 4, 11, and 14, but 2 genes on chromosomes 7, 8, 12, and 13. Such a non-random pattern of distribution indicates that specific areas of the chromosomes can be *PP2C*-enriched loci. Furthermore, a total of 5 *DlPP2C* genes were found in unanchored (UA) scaffolds, which illustrates that the genes are present in the assembled genome, but their actual chromosomal positions were not determined (Figure 2). Comprehensively, chromosomal localization analysis offers important data that lead to the understanding of the genomic organization of the *DIPP2C* gene family. The difference in the number and distribution of the genes on the chromosomes may reflect lineage-specific expansion patterns that may either be related to evolutionary adaptation or the functional specialty of *PP2C* genes in longan. The comprehensive details are shown in Supplementary Table S1, showing chromosomes, gene count, and gene density.

### 2.4. Synteny Analysis and Chromosomal Duplication of DlPP2C Genes

**Figure 3:**
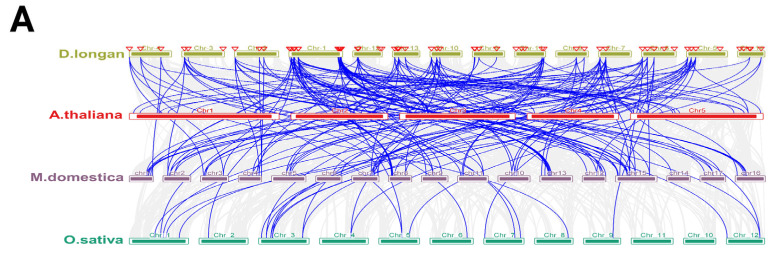

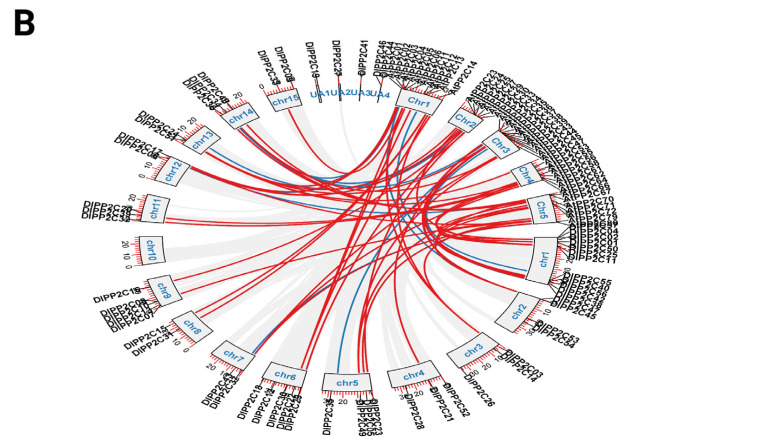
(**A**) The synteny plot of the chromosomal localization of *Arabidopsis thaliana*, *Dimocarpus longan*, *Malus domestica* Borkh, and *Oryza sativa*. The blue lines represent the synteny between *Arabidopsis* and *Longan*, and the red lines mark the syntenic associations between *Longan* and *Oryza sativa*. The map highlights the preserved gene arrangement and genomic rearrangements among the species, and provides information on their evolutionary association and the maintenance of the genomic regions throughout evolution. (**B**) The Circos map of the locus of the *PP2C* genes in the chromosomes of *Longan* (denoted as “chr”) and *Arabidopsis* (denoted as “Chr”). The red lines represent the *PP2C* genes of Longan, and the blue lines are the *PP2C* genes of *Arabidopsis*. The unanchored regions (denoted as UA1, UA2, etc.) refer to loci of the chromosomes, which could have additional *PP2C* genes, but with diminished resolution.

In order to further evaluate the pattern of evolutionary conservation and duplication of the *DlPP2C* gene family, synteny analysis was conducted to compare *Dimocarpus longan*, *Malus domestica* Borkh, and the model plant representatives of dicot and monocot species, *Arabidopsis thaliana* and *Oryza sativa* (Figure 3). This analysis demonstrated the presence of massive collinearity among the *DlPP2C* genes in the longan genome, with many intrachromosomal relationships (Figure 3A). The majority of the duplicated *DlPP2C* gene copies occurred on other chromosomes; thus, it is possible that segmental duplication or whole-genome duplication (WGD) might be the major driving force behind the expansion of the *PP2C* gene family in *longan*. Conversely, the number of duplicated gene pairs in close proximity was few, which is another sign that tandem duplication has been a rather minor process. Interestingly, chromosome 9 showed the highest number of collinearity interactions with chromosomes 5, 7, and 8, and so it is possible that the chromosome might be a hot spot of *PP2C* gene expansion. Also, multiple *DlPP2C* genes were found within unanchored (UA) genomic regions. But even though these sequences are not yet attached to particular chromosomes, their existence indicates that the sequences can add to the general diversity of the *PP2C* gene family. Incomplete genome assembly or structural complexity may also hide the true picture of the spread of the *PP2C* gene because similar associations with unanchored regions were also found in *Arabidopsis thaliana* (Figure 3B). Overall, the synteny analysis demonstrates the role of the events of segmental and whole-genome duplication in the development and evolution of the *DlPP2C* gene family. The existing results can provide meaningful information about the evolutionary dynamics and structural preservation of *PP2C* genes in the plant species.

### 2.5. Gene Structure and Conserved Motifs and Domains Analysis of the DlPP2C Gene Family

**Figure 4:**
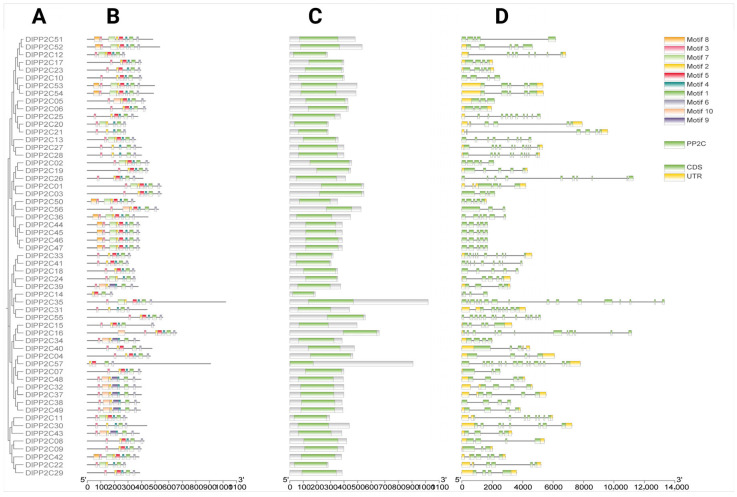
Illustration of a four-part study (from left to right) of the *DIPP2C* gene family, including evolutionary connections (**A**) phylogenetic tree, motif distributions (**B**), conserved domains (**C**), and coding sequences (CDS) and untranslated regions (UTRs) (**D**), highlighting the structural and functional diversity of the *DlPP2C* gene family.

Combined phylogenetic, conserved motif, domain analysis, and gene structure analysis allowed further analysis of the structural and evolutionary associations of the *DIPP2C* gene family (Figure 4). The conserved motif distribution analysis showed that proteins of *DIPP2Cs* have some common motifs that are similar to the conserved functional regions and are necessary for catalytic activity. It is also noteworthy that members of the same evolutionary subgroup had similar motif compositions, which indicated functional preservation, but the differences in the motif arrangement among subgroups could suggest a functional divergence. Domain analysis also revealed that all DlPP2C proteins have conserved *PP2C* catalytic domains, which are critical in their phosphatase activity. These conservation domains in the family of genes emphasize their central role in conserving core biochemical functions, and minor structural differences could help to explain the differences in substrate specificity or regulatory interactions. Analysis of the gene structure reveals the arrangement of the coding sequences (CDS) and untranslated regions (UTRs) in *DIPP2C* genes. Regions associated with protein encoding (the CDS regions) had patterns that were relatively conserved across highly related genes. Conversely, it was found that the length and structure of UTRs varied, and this can lead to post-transcriptional regulation, such as mRNA stability and translational efficiency. Taken as a whole, the homogeneity in the structure of genes among subgroups suggests both functionality and diversification in the *DlPP2C* family.

### 2.6. Protein–Protein Interaction (PPI) Analysis of PP2C Gene Family in Longan

**Figure 5:**
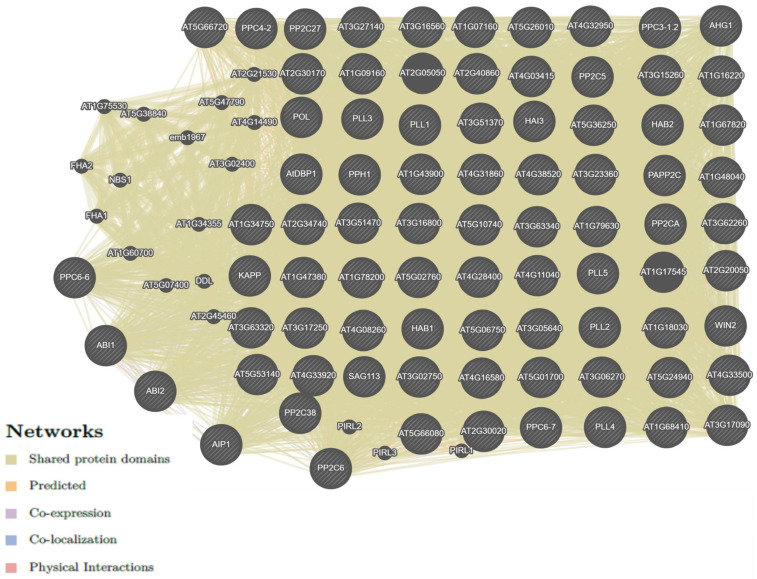
The hypothetical protein–protein interaction network model of *DlPP2C* gene family members displaying functional associations based on *Arabidopsis thaliana* as query. Each node stands for a protein, whereas edges represent predicted interactions.

In order to investigate the potential functional regulatory networks involved in DlPP2Cs, a protein–protein interaction (PPI) network was built, based on *Arabidopsis thaliana* orthologous proteins via the online database GeneMANIA (https://genemania.org) (accessed on 1 May 2026) using default parameters (Figure 5). The corresponding DlPP2C proteins, which have high similarity to those of Arabidopsis thaliana, were denoted as STRING proteins, while each individual node represents all the proteins relevant to a specific protein-coding gene locus. The PPI network shows a complex feature, and some of the DIPP2C proteins provide broad connectivity. It is noteworthy that *DlPP2C12*, *DlPP2C18*, and *DlPP2C29* have more patterns of interaction, implying that these proteins may be the key to a number of cell events. Interaction pattern annotation showed that DIPP2C proteins could be implicated in a number of biological pathways, such as signal transduction, stress response, metabolic control, and cellular homeostasis. The high connectivity of the network indicates that the DlPP2C proteins may serve as significant parts of the protein complexes in order to organize responses in the cell. A detailed report has been provided in Supplementary Table S2.

### 2.7. Cis-Acting Elements Analysis of the PP2C Gene Family in D. longan

**Figure 6:**
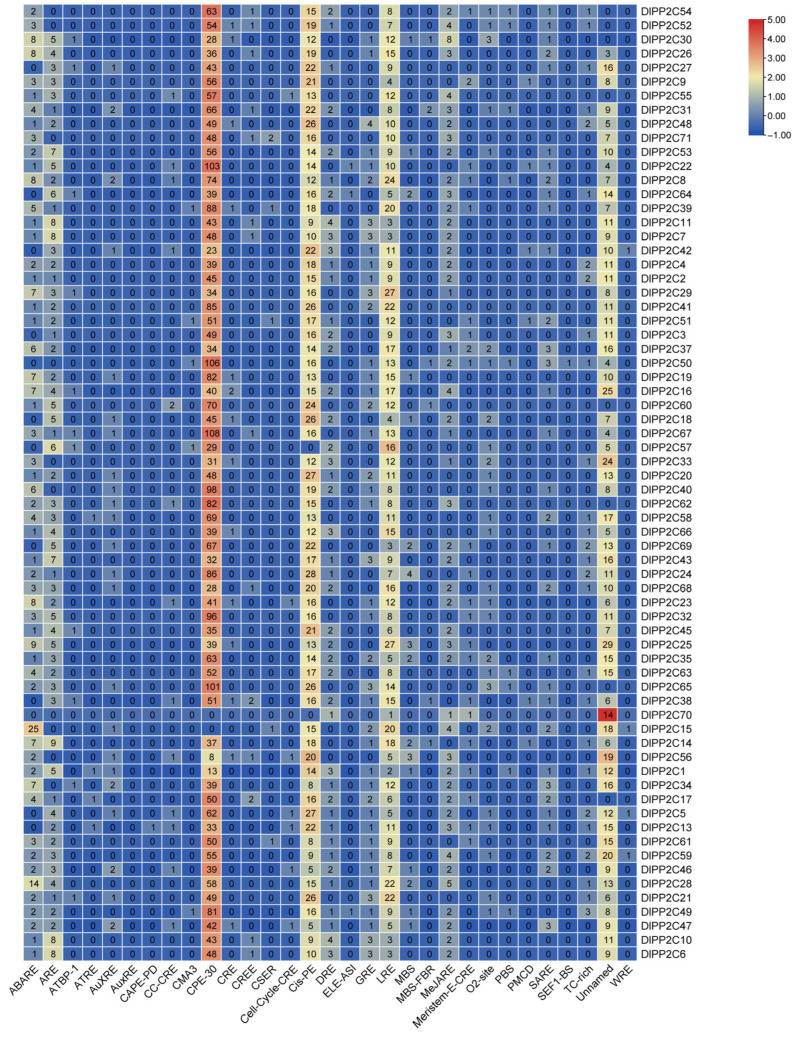
*Cis*-acting element analysis of *PP2C* genes in longan. The 2 KB promoter sequences upstream of the *DlPP2C* initiation codon (ATG) of 71 *DlPP2C* genes were analyzed with PlantCARE v1 software.

In an attempt to understand the possible transcriptional regulation of the *DlPP2C* gene family, 2000 bp upstream promoter regions have been studied in order to identify putative *cis*-acting regulatory factors (Supplementary Table S1). The promoters of *DlPP2C* genes had a large number of *cis*-elements linked to hormone responsiveness, stress responses, and developmental regulation. These included hormone-responsive elements, including abscisic acid-responsive elements (ABREs) and auxin-responsive elements (AuxREs). Specifically, the abundance of ABRE motifs suggests that many genes of DlPP2C participate in ABA-dependent processes. A higher number of CPE-30, which is a cytoplasmic binding element, was clearly seen in our findings (Figure 6). Moreover, various stress-dependent *cis*-elements were highly distributed, such as dehydration-responsive element (DRE), MYB binding sites (MBS-FBR), TC-rich repeats, MeJA-responsive element (MeJARE), and wound-responsive element (WRE). These findings suggest that *DlPP2C* genes can be linked to responses to abiotic stresses, such as drought, salinity, and oxidative stress, and biotic stress-related signaling (Supplementary Table S3). Furthermore, the promoters contained several *cis*-elements associated with developmental and cellular regulation, including CMA3, SEF1-BS, PMCD, CRE, and CC-CRE, and thus suggested their roles in growth, tissue differentiation, and circadian regulation. It is interesting to note that genes with similar *cis*-element composition tended to cluster in certain phylogenetic subsets, suggesting that transcription was regulated. By and large, the analysis of *cis*-regulatory elements indicates that the *DlPP2C* genes have complex regulatory structures that allow them to combine hormonal and environmental cues. These functional predictions, however, are found through the analysis of promoters in silico and need further experimental verification.

### 2.8. Expression Analysis of the DlPP2C Gene Family in Response to Exogenous ABA Treatment

**Figure 7:**
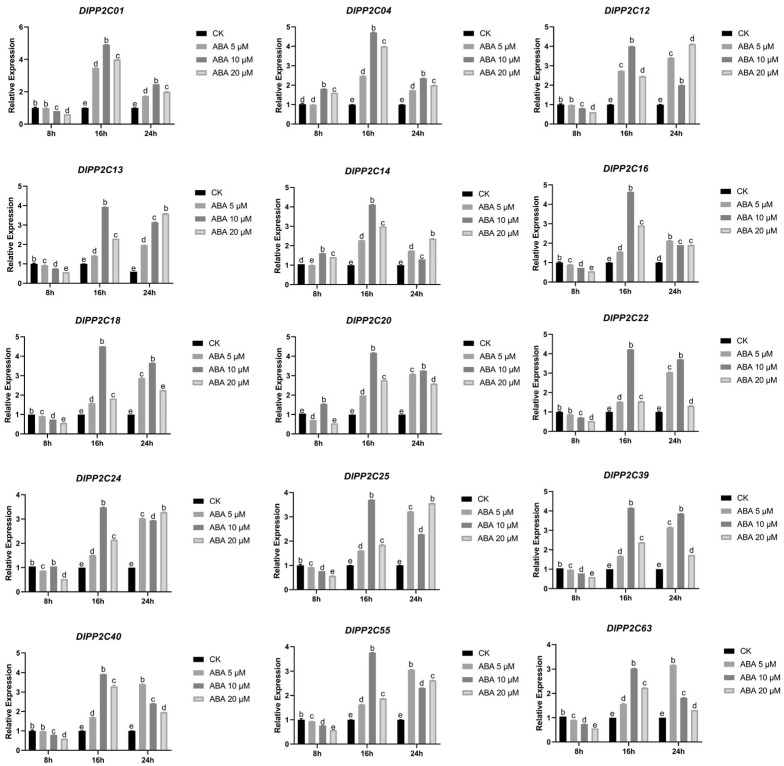
The relative expression of *DlPP2C* genes in response to exogenous ABA treatments (5 µM,10 µM, and 20 µM) at three time intervals (8 h, 16 h, and 24 h) during early SE of longan, determined by qRT-PCR. Values are the mean (*n* = 3) of three biological replicates. The data is plotted as fold change (2^−ΔΔCT^) relative to control, normalized to UBQ as internal reference. Graphpad Prism 8 (v 10.0.0) software was used for plotting the graphs using one-way ANOVA. Different letters above the bars indicate significant differences among treatments based on Tukey’s *t*-test (*p* ≤ 0.05).

The heatmap demonstrates the expression profile of the *DlPP2C* genes after ABA (Abscisic Acid) treatment. A number of genes, such as *DlPP2C1* and *DlPP2C25*, are highly expressed, which means that they are tangled in stress or response pathways mediated by ABA (Supplementary Figure S1). On the other hand, other genes like *DlPP2C28* and *DlPP2C38* show lower levels of expression, implying that they are not so imperative in ABA signaling. The study of the effect of *DlPP2C* genes during ABA signal response was performed using longan embryogenic callus subjected to various concentrations of abscisic acid (5 µM, 10 µM, and 20 µM) at different time intervals (8 h, 16 h, and 24 h). The transcriptional response of the genes of interest (*DlPP2C*) to ABA treatment was then evaluated through RT-qPCR expression of the gene. The results showed that the selected *DlPP2C* genes displayed dynamic and dose-dependent patterns of expression in response to exogenous ABA treatment. There were many genes that showed significant changes in transcription under various treatment conditions, indicating that they are sensitive to ABA concentration and duration of exposure. Interestingly, ABA treatment of 16 h at 10 µM concentration triggered the most significant levels of several *DlPP2C* gene expression, indicating that intermediate levels of concentration and intermediate exposure time are the most effective for stimulating a *PP2C*-mediated signaling response. In particular, *DlPP2C1* had a strong and consistent response to ABA treatment, with its expression being significantly different at various time points, implying its possible involvement as a key regulator in ABA signaling (Figure 7).

**Figure 8:**
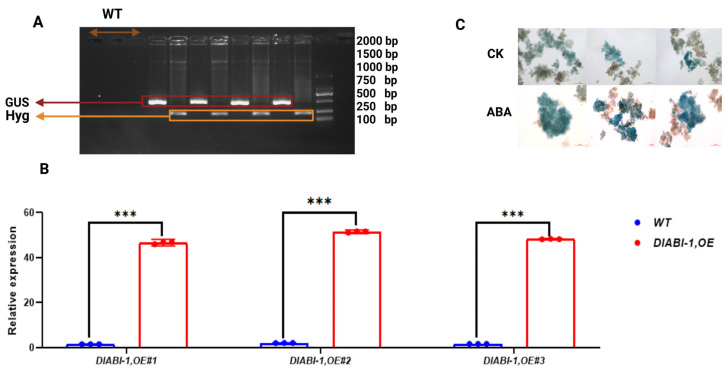
Transient overexpression of *D. longan* EC and molecular identification. (**A**) PCR amplification of WT and DlPP2C1 transient overexpression cell lines displaying the GUS (225 bp) and Hyg (475 bp), and detection for the verification of amplified sequences. (**B**) Relative expression level of WT and DlPP2C1 transient overexpression cell line determined by qRT-PCR. Asterisks indicate significant differences (WT vs. OE comparison), “***” is *p* < 0.001. (**C**) GUS staining of WT and *DlPP2C1* transiently overexpressed line. The fluorescence microscopy technique was used for capturing images with field of view set at 20×.

This was also confirmed by GUS staining of the *DlPP2C1* line of overexpression, which exhibited increased activity in the presence of ABA, indicating its efficient role in ABA-responsive regulation (Figure 8). Moreover, other *DlPP2C* genes demonstrated varied functions with respect to expression; some were prematurely induced at 8h and gradually decreased with the time interval (16–24 h), which indicates functional separation among the genes in the gene family. The difference in expression patterns of *DlPP2C* members revealed that they might have different functions in regulation during ABA signaling.

### 2.9. Weighted Gene Co-Expression Network (WGCNA) Analysis

**Figure 9:**
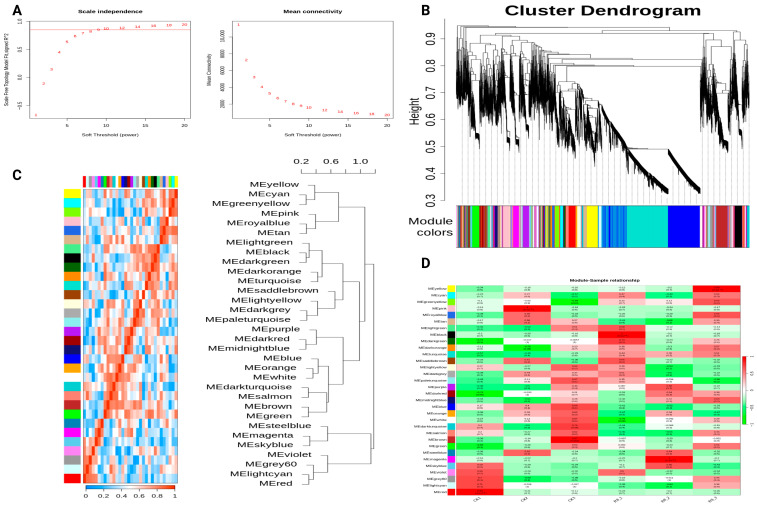
WGCNA (weighted gene co-expression network analysis) analysis. (**A**) Soft threshold selection. The scale independence was shown in the right graph, while the left graph presents mean connectivity. The horizontal coordinates of the two plots represent the value of the power index. (**B**) Module hierarchical clustering with a topologically heterogeneous matrix (TOM) set up to 1, to get the corresponding matrix of genetic non-similarity (dissTOM). (**C**) The heatmap of correlation between modules. The vertical coordinates represent the degree of difference in the node; each row and column in the lower half of the graph represents a module. (**D**) Association between samples and modules. Each row in the figure represents a different gene co-expression module, each column represents a different sample (control checks—CK1, CK2, and CK3; 10 µM ABA-treated samples—R1, R2, and R3). The value represents the correlation coefficient, and distinguishes the positive and negative correlations through red and green, respectively, and the value in the parentheses is the significant *p*-value.

To further investigate the functional relationship and regulatory networks of the DlPP2C gene family, we performed WGCNA (weighted gene co-expression network analysis) rather than a DEG (differentially expressed gene) analysis or GSEA (gene set enrichment analysis) for a comprehensive gene expression study. The analysis was performed by setting soft threshold level values, known as R2 values, for power selection, as shown in the left graph of Figure 9A. Higher R2 values indicate a better fit of the model, whereas the right graph depicts the average number of connections per node, where lower values are ideal for a scaleless network.

The resulting cluster dendrogram of genes is shown in Figure 9B. A total of 32 modules were screened, and their relationships with the samples are illustrated in Figure 9C, while several identified modules were found to be correlated with key traits, including ABA responsiveness. Each row in the figure represents a different gene co-expression module, and each column represents a different phenotype. The value corresponds to the correlation coefficient, and the colors (red and green) indicate positive and negative correlations, respectively. The value in parentheses is the significant *p*-value. A total of 25 longan *PP2Cs*, including *DlPP2C1*, were found in the turquoise module, while the other DlPP2C genes are unevenly distributed in other modules (Supplementary Table S4). The heatmap clustering of different modules is presented in Figure 9C. The deeper the color of the module, the stronger the correlation and vice versa. The correlation heatmap demonstrating the module–sample correlation is presented in Figure 9D. Modules showing a strong relationship with samples may indicate significant associations and suggest the need for further functional studies. The network heatmap plot for all genes is shown in Supplementary Figure S2.

### 2.10. Transcriptomic Sequencing and Analysis of the DlPP2C Gene Family in Response Exogenous to ABA Treatment

**Figure 10:**
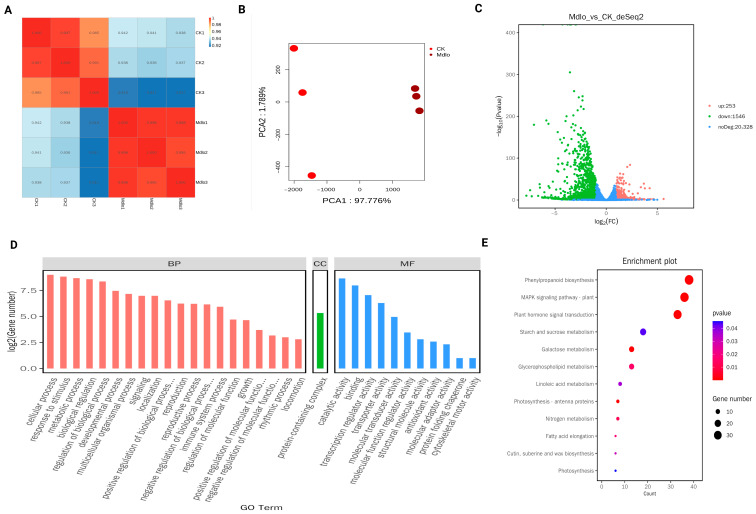
Transcriptomic analysis and data quality assessment. (**A**) Correlation heatmap between samples. A closer R2 value to 1 indicates better reproducibility between the two samples. (**B**) PC1 and PC2 are two principal components; different colors represent different groups of biological replicates. (**C**) Volcano plot on differential expression. Each dot represents a gene. X-axis: log_2_Fold change in expression; Y-axis: −log_10_ (FDR) or −log_10_ (*p*-value). (**D**) GO classification of DEGs. X-axis: GO terms and classifications; Y-axis: Number of DEGs (genes) annotated to the term (right) and percentage of that in all DEGs (genes) (Left). (**E**) KEGG pathway enrichment on DEGs-Bubble chart. Each dot represents a KEGG pathway. Y-axis: pathway; X-axis: rich factor.

Moreover, the transcriptome (RNA-seq) results of the ABA-treated and control longan EC showed vast transcriptional reprogramming. The sample correlation analysis revealed that there were high correlation coefficients between biological replicates that showed good data consistency (Figure 10A). Principal component analysis (PCA) also revealed that there was a distinct difference between control and ABA-treated groups (Figure 10B), indicating that ABA treatment may have caused a considerable amount of transcriptional change. A total of 1799 differentially expressed genes (DEGs) were identified in the course of differential expression (DEA) analysis, comprising 253 upregulated and 1546 downregulated genes (Figure 10C). The over-representation of downregulated genes shows that ABA treatment induces widespread transcriptional repression in longan callus. To further investigate the idea of the functional significance of these DEGs, Gene Ontology (GO) enrichment analysis was conducted (Figure 10D), and the enrichment in biological process was significantly related to stress response, metabolic regulation, and cellular process. In addition, KEGG pathway enrichment analysis (Figure 10E) indicated that the DEGs mainly participated in pathways of phenylpropanoid biosynthesis, MAPK signaling, and plant hormone signal transduction, respectively. The details of the individual genes involved in each pathway can be found in Supplementary Table S5.

The RNA-seq findings were in line with the RT-qPCR results, which indicate that *DlPP2C* genes are involved in the regulation of ABA-responsive transcriptional networks. Collectively, these findings postulate the vitality of the ABA-induced *DlPP2C* genes that are of prime importance in the coordination of ABA-induced gene expression and ABA-induced stress adaptation in *longan* callus. The validation of RNA sequencing data was done by performing RT-qPCR analysis on selected up-regulated and down-regulated genes, which shows that these genes were generally consistent with the transcriptomic data, indicating high reliability (Supplementary Figure S3).

## 3. Discussion

This study presents the first thorough genome-wide analysis of the *PP2C* gene family in Dimocarpus longan, a tropical fruit species of enormous economic and therapeutic relevance. Identifying 71 *PP2C* genes offers vital insights into their probable functions in regulating *Longan’s* growth, development, and stress responses [23,24]. Phylogenetic analysis showed that longan *PP2C* genes are organized into thirteen primary clades (A–L), in a way comparable to *Arabidopsis thaliana* and *Oryza sativa*, which are model dicot and monocot plants, respectively. This clustering demonstrates that the *PP2C* gene family underwent early evolutionary diversity well before the split between monocots and dicots [2,23,24]. Chromosomal mapping of the longan *PP2C* genes indicated an unequal distribution among numerous super-scaffolds, with various clusters of genes detected nearby. The clustering of *PP2C* genes across these scaffolds indicates potential gene duplication events, a primary process by which gene families increase and diversify [23,25]. Synteny’s research further confirmed the findings from the Circos plot by indicating conserved collinear areas between *Longan* and numerous other plant species, including *Arabidopsis* and *Oryza sativa* [3]. This comparative genomic study demonstrated the evolutionary and functional significance of key *PP2C* loci, supporting the concept that these genes have been conserved across numerous plant taxa [23,25]. These conserved domains demonstrate that longan *PP2Cs* share core structural properties with other PP2C proteins throughout plants [26]. Additionally, changes in exon–intron arrangement suggest that some genes may have undergone structural modifications to enable more complex control. The discovery of kinase-like domains in specific *longan PP2C* genes suggests that these genes likely participate in unique signaling pathways that are specific to this species [27]. Protein–protein interaction network analysis identified a closely connected cluster of PP2C proteins likely engaged in the abscisic acid (ABA) signaling pathway, a vital mechanism for *Longan’s* adaptation to abiotic challenges such as drought and cold stress [28]. Gene structure and conserved motif analysis of longan *PP2C* genes indicated the existence of essential functional domains, including the PP2C catalytic domain and kinase-like regions. The predominance of this cluster fits with the well-established role of *PP2Cs* as negative regulators of ABA-mediated stress responses [29]. The network analysis revealed that these *PP2Cs* are likely interacting to modify ABA signaling in a coordinated manner, which is necessary for Longan’s ability to respond to altering environmental conditions [30]. *Cis*-acting element analysis showed a variety of hormone-responsive and stress-related regulatory motifs inside the promoters of longan *PP2C* genes. Elements such as ABRE (ABA-responsive), MeJARE (methyl jasmonate-responsive), and others related to gibberellin and auxin signaling were prevalent, supporting the concept that longan PP2Cs are major integrators of numerous hormonal and environmental signals [31]. The distribution of these elements illustrated that *PP2Cs* of *D. longan* have the ability to effectively interfere with multiple signaling pathways, which allows the plants to respond to both external and internal stimuli. These *cis*-acting areas show how *PP2C* genes are regulated in response to shifting environmental factors, indicating how adaptable these genes are in facilitating adaptive reactions [32].

Abscisic acid (ABA) plays a central role in regulating plant growth, development, and stress adaptation, with clade A protein phosphatase 2C (PP2C) proteins functioning as key negative regulators in the ABA signaling pathway. In this study, several *DlPP2C* genes exhibited dynamic transcriptional responses to exogenous ABA treatment in longan embryogenic callus, with 10 μM ABA at 16 h inducing peak expression levels, particularly for *DlPP2C1*. This time- and dose-dependent response is consistent with previous findings in longan embryogenic tissues, where hormone-induced transcriptional regulation has been shown to occur in a temporally dynamic manner [33,34]. Our findings are further supported by similar studies conducted previously, where time- and dose-dependent responses were shown to be consistent in longa EC and where hormonal-induced transcriptional regulation has been shown to occur in a temporally dynamic manner [28,35,36]. *DlPP2C1*, which is ABA-responsive, may function similarly to ABI1-like *PP2Cs* in model plants. Remarkably, despite their inhibitory role at the protein level, both *ABI1* and *ABI2* genes are transcriptionally induced by ABA, establishing part of the negative feedback regulatory loop [37,38], which permits plants to fine-tune ABA signaling intensity and halt redundant responses under continued stress conditions. The expression patterns revealed in the current study, especially for *DlPP2C1*, align with this conserved regulatory model. The higher expression patterns of DlPP2C under exogenous ABA treatment, particularly at intermediate time intervals, may reflect its involvement in feedback diminution of ABA signaling [24]. This claim was further supported by the preliminary findings of GUS staining, where the higher GUS activity in the *DlPP2C1* OE line under ABA treatment was recorded, which indicates that the gene is transcriptionally activated in response to ABA. In addition to expression and introductory functional validation, RNA sequence analysis revealed an extensive transcriptional reprogramming in response to exogenous ABA treatment, with a predominance of downregulated genes. This universal repression pattern suggests that ABA may primarily suppress growth-related metabolic processes while triggering a subset of stress-responsive pathways. Analogous reports have already been published in other plant systems, where ABA induces a large-scale reorganization of gene expression to aid stress adaptation [39,40].

## 4. Materials and Methods

### 4.1. Identification and Physicochemical Properties of DlPP2C Proteins

The protein sequences of *Arabidopsis thaliana PP2C* family members were obtained from TAIR (https://www.arabidopsis.org/), (accessed on 17 March 2026) [41], while the rice PP2C sequences were retrieved EnsemblPlants (https://plants.ensembl.org/), (accessed on 17 March 2026) [42]. Firstly, the *Arabidopsis thaliana* PP2C amino acid sequences were used as a query and probe to download the HHZ *D. longan* third-generation genome from the National Center for Biotechnology Information (NCBI) Sequence Read Archive (SRA) database (SRR17675476). The TBtools software (v 2.420) [43] was used for searching possible DlPP2C sequences and further screened by two-way blast at NCBI. The PP2C conserved structural domains of the screened members were reconfirmed by using the HMMER online software (https://www.ebi.ac.uk/Tools/hmmer/search/phmmer), (accessed on 17 March 2026) [44]. Finally, the preliminarily identified members were compared with the HHZ *D. longan* third-generation genome to search for any omissions and confirmed the existence of a total of 71 *DlPP2C* gene family members, which are renamed in reference to *Arabidopsis thaliana* nomenclature for the *DlPP2C* gene family. The online software ExPASy (https://web.expasy.org/protparam/), (accessed on 17 March 2026) [45] was used to determine the number of amino acids (AA), molecular weight (MW), isoelectric point (pI), instability index (II), aliphatic index (AI), and grand average of hydropathicity (GRAVY) of DlPP2C family proteins, while the subcellular localization predictions were done using WoLF PSORT (https://wolfpsort.hgc.jp/), (accessed on 17 March 2026) [46].

### 4.2. Phylogenetic Tree, Conserved Motif, and Gene Structure of DlPP2C Family Members

The evolutionary tree between *Arabidopsis thaliana*, *rice*, and *longan* (*using full-length protein sequences*) was constructed by using the maximum likelihood (ML) algorithm (bootstrap number set to 1000) with TBtools software (V 2.420), while branches with bootstrap support ≥70% were considered well supported. [47]. The online interactive software iTOL (https://itol.embl.de/), (accessed on 19 March 2026), was used to edit and visualize the phylogenetic tree [48]. The conserved motifs of DlPP2C proteins were identified using the Multiple Em for motif Elicitation (MEME) suite (http://meme-suite.org/), (accessed on 19 March 2026) [49]. The full-length amino acid sequences of DlPP2C proteins were submitted to MEME; the maximum number of motifs was set to 10, the optimum motif width ranged from 6 to 50 residues, and other parameters were kept at default settings. The coding sequences (CDS) were aligned with their corresponding genomic DNA sequences to analyze exon–intron organization of DlPP2C genes, while the diagrams were generated using the Gene Structure Display Server (GSDS V 2.0) (http://gsds.gao-lab.org/), (accessed on 19 March 2026) [50].

### 4.3. PP2C Gene Clustering, Distribution of Chromosomes, Cis-Elements Analysis, and Synteny Visualization of DlPP2C Genes

The genome annotation (GFF/GTF) information files of *Dimocarpus longan* were used to determine chromosomal distribution of *DlPP2C* genes. The TBtools software (version 2.420) was utilized for mapping *DlPP2C* genes onto their respective chromosomes. PlantCARE (http://bioinformatics.psb.ugent.be/webtools/plantcare/html/), (accessed on 19 March 2026), was used to analyze the *cis*-acting element prediction [51]. For synteny analysis, MCScanX analysis between *D. longan*, *Arabidopsis thaliana* (L.) Heynh., and *Oryza sativa* subsp. *japonica Kato*, with an E-value threshold of 1e^−3^, was performed using the TBtools software v 2.420 (https://github.com/CJ-Chen/TBtools-II, accessed on 28 March 2026).

### 4.4. Protein–Protein (PPI) Interaction Network Analysis

The GeneMANIA database was used to predict the potential interaction relationships of DlPP2C proteins [52]. The DlPP2C protein sequences were first used to identify homologous proteins in *Arabidopsis thaliana* due to the limited annotation of *Dimocarpus longan*.

### 4.5. Plant Materials, ABA Treatments, and Expression Analysis

For the current study, we used embryogenic callus (EC) of *D. longan* Lour-Honghezi. Samples of 0.2 g EC were treated with ABA in MS medium provided by Coolaber manufacturer, Beijing city, China, after 20 days of proliferation at concentrations of 5 μM, 10 μM, and 20 μM. Treated materials were incubated in a dark environment at 25 °C for three time intervals of 8 h, 16 h, and 24 h. EC in MS medium without ABA treatment was used as a control. For each treatment, three biological replicates were performed, and samples were collected and frozen in liquid nitrogen and stored at −80 °C in a refrigerator.

### 4.6. The qRT-PCR Analysis

The TransZol Up kit provided by TransGen (Beijing, China) was used for the total RNA extraction from samples following the instruction manual. The cDNA synthesis was completed using Revertaid Master Mix (Thermo Fisher Scientific, Shanghai, China), and qRT-PCR was performed on the Roche Light Cycler 96 instrument with 10-fold diluted cDNA as an amplification template. UBIQUITIN (UBQ) was used as an internal reference [53,54]. Data calculations were performed according to 2^−ΔΔCt^ [54], and graphs were generated using GraphPad Prism 8.0.2 software. DNAMAN 6.0 software was used to design the qRT-PCR primers (Supplementary Table S6).

### 4.7. RNA Sequencing and Analysis

Based on the comprehensive assessment and expression outcomes, which aimed to capture the most biologically informative, reproducible, and interpretable transcriptomic response, the samples treated with 10 µM ABA (labeled as Mdlo) for 16 h and a control group (CK) with three independent biological replicates were subjected to RNA sequencing analysis (Biomarker Biotechnology Co., Ltd., Beijing, China). Total RNA was sequenced for the six groups of longan embryogenic cultures. The purity and concentration of RNA were assessed using a Nanodrop 2000 spectrophotometer, and RNA integrity was verified using the Agilent 2100/Lab Chip GX [54,55,56]. After samples passed quality control, library construction and mRNA transcriptome sequencing were carried out. HISAT2 (Hierarchical Indexing for Spliced Alignment of Transcripts) software was used to quickly and accurately compare the clean reads with the reference genome, and to acquire corresponding information for the reads on the reference genome [57]. The reads were then assembled using String Tie to reconstruct the transcriptome for subsequent analysis [58]. The genes were annotated for various analyses, including DEGs and KEGG [59]. The thresholds of |log2(fold change)| > 1 and FDR < 0.01 were used to filter DEGs. GO and bioinformatics analysis were conducted according to the methods described as follows [60]. The weighted gene co-expression network analysis (WGCNA) was performed using the R package (version 1.74). The data from the ABA treatment for 16 h at 10 µM and the control were imported into the WGCNA package. The correlation-based associations between gene modules were set to 15 and calculated, and analysis was performed using default settings.

### 4.8. Transient Transformation of D. longan Embryogenic Callus

The DNAMAN 9.0 software was used to design the specific amplification primers at the 3′ and 5 ends of the *Dlo000068* (*DlPP2C1*) CDS sequence, which were cloned into the pCAMBIA1301-35-GUS vector (Supplementary Table S7). The bacterial solution comprising the recombinant plasmid was activated, and cells were gathered by centrifugation at 7800 r/min for a duration of 10 min. The collected cells were further resuspended by using an MS suspension medium containing 30 g/L sucrose, 200 mM AS, and 100 mM MgCl_2_ in the infiltration solution. The OD_600_ was adjusted between 0.6 and 0.8. The 15-day-old *D. longan* EC was co-cultured with Rhizobium radiobacter for 30 min; the sap was then filtered and transferred to MS solid media for 3 d. The wild type (WT), having an empty vector, and the pCAMBIA1301-35-GUS were taken as control checks, whereas transient overexpression DlPP2C cell lines were labeled as *DlPP2C*1-OE#1,2,3.

### 4.9. GUS Staining and PCR Amplification

A measure of 0.1 g each of the transiently transformed pCAMBIA1301 and pCAAMBIA1301:DlPP2C1: GUS(OE1-OE3) cell lines was collected. After freezing in liquid nitrogen, the samples were stored in −80 °C refrigerator for further analysis. Following the instructions mentioned on the GUS staining kit provided by Huayueyang Biotechnology, China, the GUS staining on the transgenic materials was completed. The fluorescence microscope (LEICA DMI8, Wetzlar, Germany) was used to obtain and observe the images of the GUS-stained *D. longan* EC transgenic cells under a 20x field of view. For the DNA extraction from both WT and overexpression cell lines after transient transformation, the Plant Genomic DNA Kit (ThermoFisher, Waltham, MA, USA) was used. PCR amplification using F/R primers of GUS and Hyg (Supplementary Table S3) was used for the identification of transgenic cell lines.

## 5. Conclusions

Our findings provide a comprehensive exploration of the *PP2C* gene family in *Dimocarpus longan* during early somatic embryogenesis. In the context of ABA signaling, both during development processes and under stress conditions, *DlPP2C1* (*ABI1*) shows a significant response to ABA, which suggests that *DlPP2C1* is an ABA-responsive regulator that influences gene expression in response to ABA. The uniformity between RNA-seq data and RT-qPCR findings strengthens the suggestion of the involvement of *DlPP2C1* in ABA-mediated regulatory networks, but further studies are required to confirm whether it acts as a negative regulator or functions in another capacity within the ABA signaling cascade. Taken together, the evidence from ABA treatment assays, GUS staining findings, and transcriptomic outcomes suggests that *DlPP2C1* responds to ABA and participates in ABA-mediated signaling during early somatic embryogenesis in *Dimocarpus longan*. Overall, our results provide new insights into the functional role of *DlPP2C* genes in ABA signaling, establishing a basis for future investigations into their roles in stress adaptation and application to other important plant species, particularly those with challenging micropropagation systems.

## Figures and Tables

**Table 1:** Physicochemical properties of the *PP2C* gene family in *D. longan*. Columns detail the gene IDs list, number of amino acids (A.A), molecular weight (MW), isoelectric points (pI), instability index (II), aliphatic index (AI), grand average of hydropathicity (GRAVY), and predicted subcellular localization (SCL).

ID	AA	MW	pI	II	AI	GRAVY	SCL
DlPP2C1	481	53,095.49	5.37	46.39	68.07	−0.469	Chlo
DlPP2C2	487	53,534.05	5.63	41.3	76.28	−0.437	Chlo
DlPP2C3	532	58,738.96	4.95	38.69	81.43	−0.367	Cito
DlPP2C4	494	54,353.02	5.53	40.61	78.16	−0.403	Chlo
DlPP2C5	446	50,102.44	6.29	31.26	75.61	−0.579	Cyto
DlPP2C6	386	42,984.75	6.36	40.35	76.53	−0.279	Chlo
DlPP2C7	386	42,984.75	6.36	40.35	76.53	−0.279	Chlo
DlPP2C8	380	41,976.53	7.99	48.26	81.08	−0.31	Chlo
DlPP2C9	351	38,552.76	5.71	44.38	88.29	−0.273	Cyto
DlPP2C10	386	42,904.35	5.28	41.16	76.04	−0.299	Mito
DlPP2C11	386	42,904.35	5.28	41.16	76.04	−0.299	Mito
DlPP2C12	713	80,002.69	5.44	43.35	78.49	−0.517	Nucl
DlPP2C13	709	79,381.33	5.41	45.21	73.96	−0.601	Nucl
DlPP2C14	397	42,739.4	7.53	60.46	76.12	−0.28	Chlo
DlPP2C15	431	47,829.8	5.97	61.46	65.57	−0.581	Cyto
DlPP2C16	427	46,665.56	5.6	58.58	77.82	−0.417	Nucl
DlPP2C17	786	86,793.15	5.36	42.48	79.21	−0.474	Chlo
DlPP2C18	882	98,099.08	5.93	39.87	68.21	−0.56	Nucl
DlPP2C19	393	42,503.3	6.38	44.89	83.77	−0.188	Chlo
DlPP2C20	351	38,708.11	5.84	36.22	86.58	−0.329	Nucl
DlPP2C21	448	49,649.68	8.24	36.49	85.87	−0.376	Mito
DlPP2C22	524	57,090.5	5.17	47.32	88.95	−0.188	Nucl
DlPP2C23	397	43,513.15	5.88	46.48	85.31	−0.359	Chlo
DlPP2C24	494	54,248.02	8.59	44.14	77.91	−0.43	Nucl
DlPP2C25	418	45,486.45	5.56	62.73	81.2	−0.278	Nucl
DlPP2C26	455	50,300.21	6.12	49.41	83.54	−0.415	Chlo
DlPP2C27	544	59,008.92	4.89	44.04	91.69	−0.12	Chlo
DlPP2C28	397	43,225.38	4.96	55.72	74.84	−0.434	Nucl
DlPP2C29	546	58,749.44	4.9	47.91	93.35	−0.167	Chlo
DlPP2C30	464	51,427.31	5.51	65.62	82.28	−0.258	Chlo
DlPP2C31	293	31,518.87	5.05	39.49	77.92	−0.327	Cyto
DlPP2C32	356	39,302.94	5.24	47.5	75.06	−0.4	Chlo
DlPP2C33	276	30,273.96	5.1	30.36	82.28	−0.244	Cyto
DlPP2C34	353	39,479.4	8.91	42.23	91.42	−0.219	Chlo
DlPP2C35	658	73,053.46	6.11	40.91	93.31	−0.187	Cyto
DlPP2C36	282	31,023.03	7.76	40.74	82.98	−0.413	Cyto
DlPP2C37	282	31,011.19	5.68	42.61	89.18	−0.296	Cyto
DlPP2C38	283	30,887.89	6.75	38.41	82.37	−0.385	Nucl
DlPP2C39	406	44,250.34	5.09	53.3	89.11	−0.058	Nucl
DlPP2C40	394	42,888.75	5.87	49.59	80.96	−0.307	Nucl
DlPP2C41	187	20,712.63	6.15	38.64	101.12	−0.14	Chlo
DlPP2C42	428	45,737.3	6.9	32.34	86.33	−0.111	Chlo
DlPP2C43	439	47,181.98	8.24	36.67	86.86	−0.174	Chlo
DlPP2C44	438	47,673.46	5.17	37.87	82.58	−0.193	Cyto
DlPP2C45	400	44,478.72	8.15	57.04	77.5	−0.357	Chlo
DlPP2C46	318	35,003.53	8.21	43.44	83.43	−0.393	Cyto
DlPP2C47	303	33,325.7	8.22	43.19	84.65	−0.364	Cyto
DlPP2C48	277	31,547.17	9.68	55.24	75.63	−0.467	Chlo
DlPP2C49	398	43,428.37	5.76	37.62	93.87	−0.114	Chlo
DlPP2C50	398	43,331.26	5.76	37.45	91.91	−0.129	Chlo
DlPP2C51	410	44,560.11	5.07	35.52	79.98	−0.348	Chlo
DlPP2C52	907	100,000.67	4.83	46.17	96.37	−0.09	Plas
DlPP2C53	476	52,456.31	5.7	39.43	82.23	−0.214	Chlo
DlPP2C54	155	17,163.21	6.81	36.12	62.32	−0.517	Nucl
DlPP2C55	372	41,033.06	5.44	41.47	71.34	−0.422	Nucl
DlPP2C56	306	32,961.16	5.05	39.31	92.06	0.013	Chlo
DlPP2C57	514	55,360.65	6.23	44.45	80.04	−0.227	Mito
DlPP2C58	453	47,908.98	8.48	40.16	78.98	−0.09	Chlo
DlPP2C59	394	44,304.57	6.9	50.54	96.24	−0.199	Mito
DlPP2C60	391	43,315.21	5.85	37.44	94.99	−0.207	Cyto
DlPP2C61	385	43,139.22	8.12	42.64	90.34	−0.288	Chlo
DlPP2C62	397	44,158.32	8.67	48.55	88.84	−0.307	Chlo
DlPP2C63	397	44,176.3	8.94	48.68	90.3	−0.272	Nucl
DlPP2C64	385	42,838.69	7	38.99	91.14	−0.289	Chlo
DlPP2C65	374	41,591.6	8.86	43.57	87.35	−0.293	Mito
DlPP2C66	383	42,310.97	5.74	42.23	89.32	−0.193	Chlo
DlPP2C67	1018	114,972.38	5.62	45.74	80.42	−0.382	Vacu
DlPP2C68	243	26,329.38	9	42.67	98.35	−0.016	Cyto
DlPP2C69	258	28,459.06	4.9	42.67	79.73	−0.252	Extr
DlPP2C70	454	48,976.75	4.72	42.62	88.24	−0.137	Chlo
DlPP2C71	555	61,220.53	5.55	48.62	87.48	−0.235	Chlo

## Data Availability

All relevant data are available within the manuscript and the Supplementary Materials.

## Supplementary Materials

The following supporting information can be downloaded at https://www.mdpi.com/article/10.3390/plants15111659/s1: Supplementary Figure S1. The heat map generated from the RNA sequencing FPKM values for DlPP2C genes with TBtools software v2.080 software under the ABA treatment of 16 h at 10 µM mM concentration. The control denoted with (CK) whereas ABA treated samples renamed as (Mdlo). Different colors on the scale bar represent different transcript levels. The data shown in the figure are the original FPKM value, with two decimal places selected. Supplementary Figure S2. The modular gene cluster heat map generated from the WGCNA (weighted gene co-expression network analysis). Each row and column represents a gene, and the darker the color of each point (white->yellow->red) represents the stronger the connectivity between the two genes corresponding to the row and column. Supplementary Figure S3. The relative expression of (A) Up-regulated and (B) Down-regulated *DlPP2C* genes in response to exogenous ABA treatments (10 µM) at time interval of (16 h), during early SE of longan determined by qRT-PCR. Values are the mean (*n* = 3) of three biological replicates. The data is plotted as fold change (2^−ΔΔCT^) relative to control, normalized to UBQ as internal reference. Graphpad prism 8 (v 10.0.0), software was used for plotting the graphs using one way ANOVA. Asterisks indicate significant differences (control vs. ABA treatments comparison) based on Tuckey’s *t*-test, “***” is *p* < 0.001. Supplementary Table S1: Gene count and Gene density of Chromosomes; Supplementary Table S2: The hypothetical predicted protein-protein interaction network model of *DlPP2C* gene family members displaying functional associations based on Arabidopsis thaliana as query. Each node stands for a protein whereas edges represent predicted interactions; Supplementary Table S3: *Cis*-acting element analysis of *PP2C* genes in longan. The 2 KB promoter sequences upstream of the *DlPP2C* initiation codon (ATG) of 71 *DlPP2C* genes were analyzed with PlantCARE; Supplementary Table S4: Hub of Genes for Each module; Supplementary Table S5: Genes in Each Pathway; Supplementary Table S6: Primers used; Supplementary Table S7: Vector construction primer sequences used.

## Abbreviations

The following abbreviations are used in this manuscript:

ABA	Abscisic acid
PP2C	Protein phosphatase 2C
ABI	Abscisic acid-insensitive
WGCNA	Weighted gene co-expression network analysis
GO	Gene Ontology
KEGG	Kyoto Encyclopedia of Genes and Genomes
DEGs	Differentially expressed genes
UBQ	UBIQUITIN
SRA	Sequence Read Archive
GSDS	Gene Structure Display Server
