# Supplementary figures and images for: Genome-Wide Analysis of PP2C Gene Family and Identification of DlPP2C1 as an ABA-Responsive Candidate Regulator During Early Somatic Embryogenesis in Longan (Dimocarpus longan Lour.)
Source: Plants (Basel). 2026 May 28;15(11):1659. doi: 10.3390/plants15111659 (PMC13259482; doi:10.3390/plants15111659)

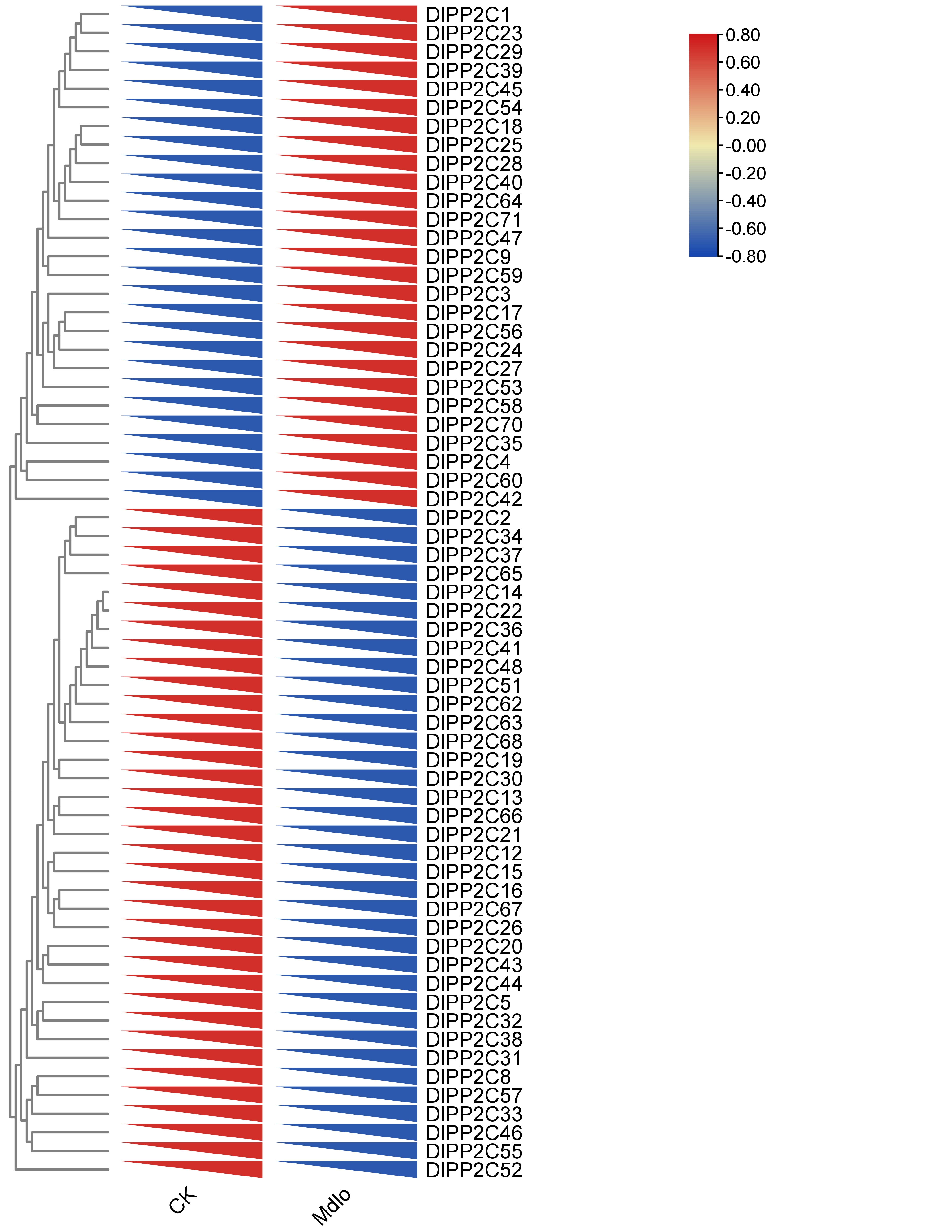

Supplement: Supplementary file 1 [file plants-15-01659-s001.zip › Supplementary Figure 1..jpg]

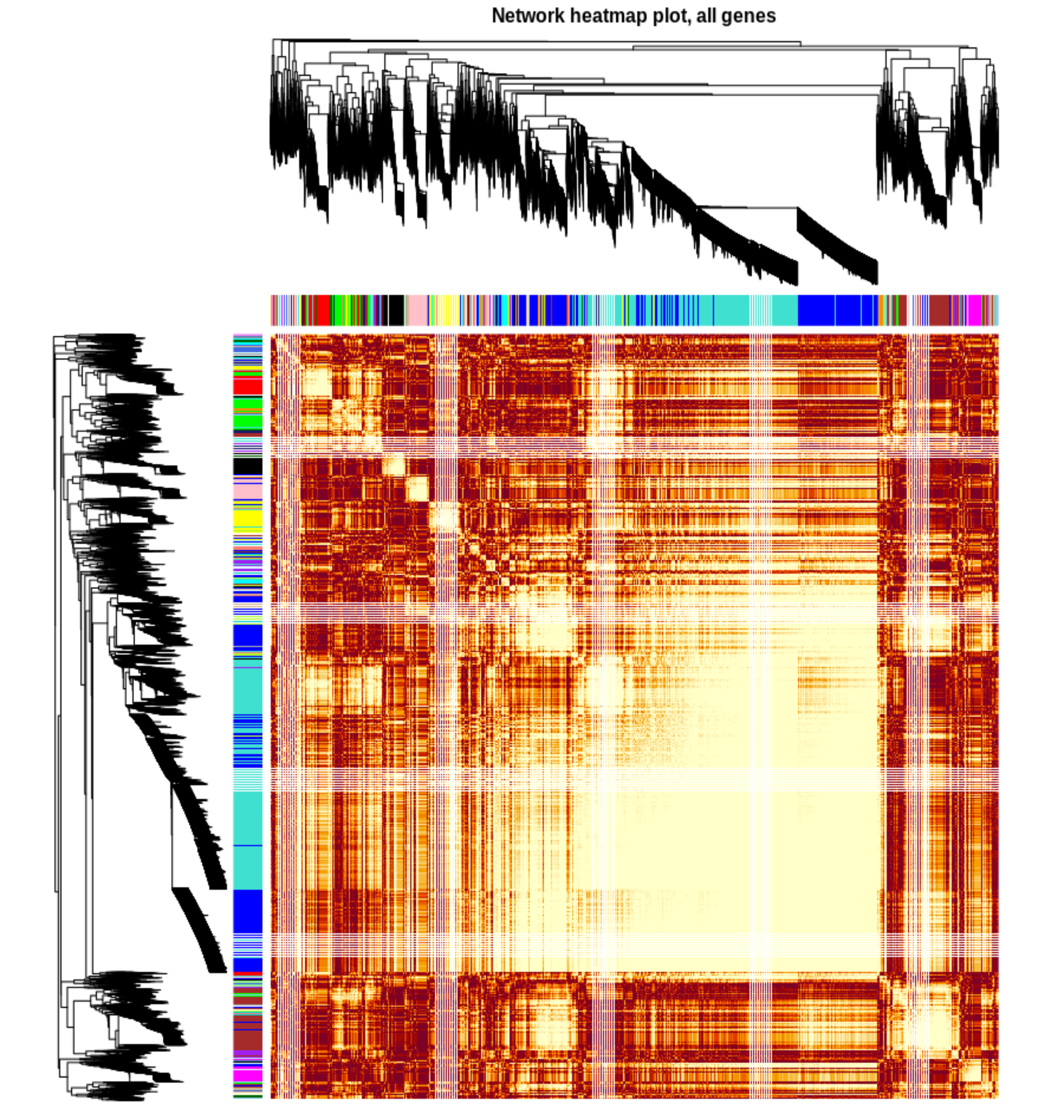

Supplement: Supplementary file 1 [file plants-15-01659-s001.zip › Supplementary Figure 2..jpeg]

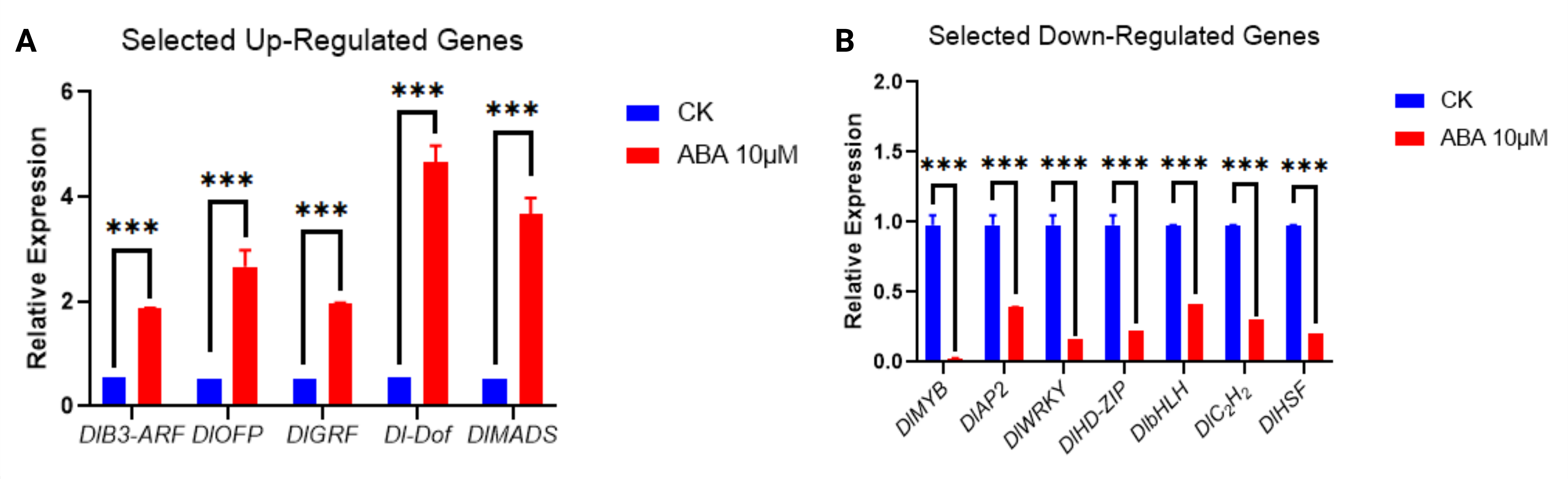

Supplement: Supplementary file 1 [file plants-15-01659-s001.zip › Supplementary Figure 3..jpeg]
